# Human Breast Extracellular Matrix Microstructures and Protein Hydrogel 3D Cultures of Mammary Epithelial Cells

**DOI:** 10.3390/cancers13225857

**Published:** 2021-11-22

**Authors:** Chandler R. Keller, Yang Hu, Kelsey F. Ruud, Anika E. VanDeen, Steve R. Martinez, Barry T. Kahn, Zhiwu Zhang, Roland K. Chen, Weimin Li

**Affiliations:** 1 Department of Translational Medicine and Physiology, Elson S. Floyd College of Medicine, Washington State University, Spokane, WA 99202, USA; chandler.keller@wsu.edu (C.R.K.); kelsey.ruud@providence.org (K.F.R.); 2 Department of Crop and Soil Sciences, College of Agricultural, Human, and Natural Resources Sciences, Washington State University, Pullman, WA 99164, USA; yang.hu@wsu.edu (Y.H.); zhiwu.zhang@wsu.edu (Z.Z.); 3 School of Mechanical and Materials Engineering, Washington State University, Pullman, WA 99164, USA; anika.vandeen@wsu.edu (A.E.V.); roland.chen@wsu.edu (R.K.C.); 4 Department of Surgery, The Everett Clinic and Providence Regional Cancer Partnership, Everett, WA 98201, USA; Steve.Martinez@everettclinic.com; 5 Department of Medical Education and Clinical Sciences, Elson S. Floyd College of Medicine, Washington State University, Spokane, WA 99202, USA; 6 CellNetix Pathology & Laboratories, Seattle, WA 98104, USA; bkahn@cellnetix.com; 7 Providence Regional Medical Center, Everett, WA 98201, USA

**Keywords:** breast tissue, extracellular matrix, structure, hydrogel, microenvironment, breast cancer, machine learning, 3D culture, acini, morphology

## Abstract

**Simple Summary:**

Human breast tissue extracellular matrix (ECM) is a microenvironment essential for the survival and biological activities of mammary epithelial cells. The ECM structural features of human breast tissues remain poorly defined. In this study, we identified the structural and mechanical properties of human normal breast and invasive ductal carcinoma tissue ECM using histological methods and atomic force microscopy. Additionally, a protein hydrogel was generated using human breast tissue ECM and defined for its microstructural features using immunofluorescence imaging and machine learning. Furthermore, we examined the three-dimensional growth of normal mammary epithelial cells or breast cancer cells cultured on the ECM protein hydrogel, where the cells exhibited biological phenotypes like those seen in native breast tissues. Our data provide novel insights into cancer cell biology, tissue microenvironment mimicry and engineering, and native tissue ECM-based biomedical and pharmaceutical applications.

**Abstract:**

Tissue extracellular matrix (ECM) is a structurally and compositionally unique microenvironment within which native cells can perform their natural biological activities. Cells grown on artificial substrata differ biologically and phenotypically from those grown within their native tissue microenvironment. Studies examining human tissue ECM structures and the biology of human tissue cells in their corresponding tissue ECM are lacking. Such investigations will improve our understanding about human pathophysiological conditions for better clinical care. We report here human normal breast tissue and invasive ductal carcinoma tissue ECM structural features. For the first time, a hydrogel was successfully fabricated using whole protein extracts of human normal breast ECM. Using immunofluorescence staining of type I collagen (Col I) and machine learning of its fibrous patterns in the polymerized human breast ECM hydrogel, we have defined the microstructural characteristics of the hydrogel and compared the microstructures with those of other native ECM hydrogels. Importantly, the ECM hydrogel supported 3D growth and cell-ECM interaction of both normal and cancerous mammary epithelial cells. This work represents further advancement toward full reconstitution of the human breast tissue microenvironment, an accomplishment that will accelerate the use of human pathophysiological tissue-derived matrices for individualized biomedical research and therapeutic development.

## 1. Introduction

The breast tissues of women dynamically change their morphologies, cellular activities, and expression of biomolecules during puberty, throughout the menstrual cycle, and peri-natal period [1]. Fluctuations of systemic or local levels of hormones and growth factors such as estrogen, progesterone, insulin, and insulin-like growth factor, contribute to these tissue changes. Under normal physiological conditions, these physical and biochemical changes do not adversely alter the basic functions and structures of the tissues [2], but are disrupted in the setting of malignancy, which results in upregulated cancer and stromal cell activities, such as transcription, translation, uncontrolled proliferation, activation of tissue resident cells, de-differentiation, altered metabolism, secretion or degradation of biomolecules, and infiltration of immune cells into the stroma with resultant destruction of the breast tissue ECM architecture [3,4,5]. Interestingly, the destructive ECM changes noted above are accompanied by excessive collagen deposition and desmoplastic tissue changes [6]. The distinction between ECM structures in human breast cancer tissues from those of normal breast tissues is poorly defined. It therefore follows that the biological phenotypes of human breast cancer cells grown in a bioengineered native human breast tissue ECM microenvironment are heretofore undefined. The goal of this study was to define the ECM microstructural features of normal human breast and invasive breast cancer tissues and examine the biological phenotypes of normal and cancerous mammary epithelial cells grown in primary human breast tissue ECM microenvironment.

Because the pathophysiological changes of the breast are complex and difficult to model and because molecular studies in the human body are impractical, we must rely on in vitro *or* ex vivo tissue-mimicking cell cultures and animal models as alternative tools to better understand human breast cancer biology and to develop novel therapeutic approaches. Among these model systems, tissue-mimicking three-dimensional (3D) cultures have become an indispensable tool for cancer research. Various 3D culture models have been fabricated using biomimetic materials, such as polyethylene glycol (PEG), polylactic-co-glycolic acid (PLGA), polycaprolactone (PCL) [7,8]. Similar 3D culture models have been developed using native biomaterials such as collagen or Matrigel as cell growth substrata [9]. Both types of materials have shown capacity to support cell survival, 3D growth, and therapeutic drug testing, as we and others have demonstrated [10,11,12]. Hybrid materials made of a combination of biomimetic materials and/or native biomaterials have been broadly used for a better approximation of the native tissue microenvironment to address specific biological or pathophysiological questions [13,14,15]. Interestingly, a general trend of utilizing biomimetic materials (or their composites) for bioengineering or drug delivery studies [16,17,18,19] and using native biomaterials for tissue culture and cell biology research has formed [20,21,22,23]. This seemingly coincidental bifurcation in the material used for different biomedical and pharmaceutical applications reflects an adaptive ‘natural selection’ process where the biomimetic materials and native biomaterials could serve their best roles in their relevant fields.

The advent of cell spheroid, tumoroid, or organoid 3D cultures in native biomaterials, such as collagen, fibrin, HA, or Matrigel (also called lrECM for laminin-rich ECM) hydrogels, has created a surge of tissue microenvironment-mimicking research [24,25,26,27], heralding more biologically relevant cancer research discoveries and improved testing of therapeutic drugs. A fundamental question remaining for these advanced models to address is how close the ‘native tissue’-derived but not ‘specific tissue’-derived culture substrata represents the original tissue microenvironment of the cells living in it. To explore a resolution of this question, we have recently generated ECM hydrogels from mouse or pig breast tissues that preserved the majority of the tissues’ ECM proteins essential for mimicking the native breast tissue microenvironment [11,28]. In addition to supporting normal or cancerous mammary epithelial cell survival and growth within a 3D space [11,28,29], the breast tissue ECM hydrogels induced the expression of epithelial cell membrane receptors and other biomarkers distinct from that of the cells grown on collagen or Matrigel. Additionally, human breast cancer cells exhibited distinguishable morphologies, differentiation, migration, invasion, and metabolic profiles when cultured on the different matrices [29,30]. These observations highlight the importance of using tissue-related culture materials for tissue-specific cell cultures. Here, we moved a step further and modeled human breast tissue microenvironment with human breast tissue ECM at both compositional and structural levels and studied human mammary epithelial cell biology using the primary tissue ECM.

We hypothesized that human breast tissue ECM is a native microenvironment suitable for human mammary epithelial cell growth and exhibition of biological phenotypes as seen in native tissues. This has been tested via a set of experiments that include comparing the ECM structural features of human normal breast and invasive breast cancer tissue ECM at both intact and decellularized tissue levels; extracting normal breast tissue ECM proteins to form hydrogel; comparing the microstructural features of the polymerized human breast tissue ECM hydrogel with those of pig breast tissue ECM hydrogel, collagen, and Matrigel using machine learning technology; and examining human normal and cancerous mammary epithelial cell 3D growth within the breast ECM hydrogel. Data gleaned from this study will provide insights into human breast tissue ECM pathology and native mammary epithelial cell spatial biology related to malignant conditions.

## 2. Materials and Methods

### 2.1. Patient Specimens

Fresh breast tissue specimens were obtained from two patients with invasive ductal carcinoma who were treated with mastectomy. Both patients provided informed consent for this study and the associated study protocol was approved by the Providence Regional Medical Center Institutional Review Board. The normal and lesion specimens were divided into portions for planned experiments: formalin fixation and paraffin embedding for histological staining; optimal cutting temperature (OCT) compound fixation and cross sectioning for stiffness measurement with atomic force microscopy (AFM); ECM extraction and hydrogel generation.

### 2.2. Human Breast Tissue ECM Extraction

The normal breast tissues were diced, homogenized in ice cold deionized water for up to 4 h, and centrifuged at 10,000 rpm for 30 min at 42 °C to remove fat and oil droplets. Then, the samples were decellularized in 1% Triton X-100 at a volume that is approximately 10-fold that of the tissue samples at RT for 5–7 days (changing the detergent solution every day), washed in deionized water 3 times for over 4 h each, and subjected to either hydrogel extraction or lyophilization for storage or formalin-fixation and paraffin-embedding for cross sectioning and staining.

### 2.3. H&E Staining

Formalin-fixed and paraffin-embedded (FFPE) breast tissue or decellularized tissue ECM samples were cross sectioned (10 μm thick) using a microtome. The cross sections on slides were deparaffinized twice in xylene for 3 min each; rehydrated sequentially in 100% ethanol for 3 min, 95% ethanol for 3 min, and deionized (DI) water for 2 min; stained with hematoxylin for 2 min, followed by rinsing with DI water for 1 min, 70% ethanol and 1% HCl for 1 min, and DI water for 1 min; stained with eosin for 30 s, followed by rinsing with DI water for 1 min, 95% ethanol for 1 min, 100% ethanol for 2 min, xylene for 4 min; and mounted with coverslips for light microscopy examination.

### 2.4. Immunofluorescence (IF) Staining of FFPE Samples

The cross sections of the FFPE specimens were deparaffinized three times in xylene for 5 min each; rehydrated in 100% ethanol for 5 min, 95% ethanol for 5 min, 70% ethanol for 5 min, and DI water for 2 min; subjected to antigen retrieval with 20 mM sodium citrate buffer containing trisodium citrate dihydrate and Tween 20 at 95 °C for 20 min, followed by cooling to room temperature (RT) and DI water washing for 5 min. The tissue sections were then permeabilized with 0.1% Triton X-100 in PBS at

RT for 20 min and blocked with 3% BSA in PBS at RT for 45 min; stained with primary antibody for Collagen I (Novus Biologicals, Centennial, CO, USA; #NB600-408) in 3% BSA at 4 °C overnight, followed by washing with 0.1% Triton X-100 in PBS, staining with Alexa Fluor 488-conjugated secondary antibody (Thermo Fisher Scientific, Waltham, MA, USA; #A-11034), washing with 0.1% Triton X-100 in PBS, staining with Hoechst (Bio-Techne, Minneapolis, MN, USA; #5117) for 5 min, and mounting coverslips. The slides were stored in the dark at 4 °C overnight and imaged under a fluorescence microscope.

### 2.5. Human Breast Tissue Matrix Gel (HB-TMG) Generation from Breast Tissue ECM

The decellularized human normal breast tissue ECM was rehydrated with ddH_2_O in a test tube, centrifuged at 10,000 rpm for 5 min to remove the water, flash frozen in liquid nitrogen, cut into small pieces and ground in liquid nitrogen in a mortar. The pulverized ECM was collected into a 2 mL test tube, washed twice in 3.4 M NaCl buffer (99.25 g NaCl, 6.25 mL of 2 M Tris Base, 0.75 g of EDTA, and 500 mL of ddH_2_O, pH 7.4) at 4 °C for 15 min each, pelleted by centrifugation, and subject to protein extraction in a series of urea and thiourea solutions: 2 M urea buffer (1.2 g urea, 0.0605 g Tris HCl, and 0.09 g NaCl in 10 mL of ddH_2_O, pH 8.0), rotating in 4 °C overnight, collecting the protein extract by centrifugation at 15,000 rpm and 4 °C for 30 min, and storing the extract on ice; The previous steps were repeated with 4 M urea, 6 M urea, 6 M/0.5 M urea/thiourea, 6 M/2 M urea/thiourea, 7 M/0.5 M urea/thiourea, 7 M/2 M urea/thiourea, 8 M urea, and 2% n-octyl β-D-glucopyranoside on the remaining insoluble ECM. The protein extracts in the urea/thiourea solutions were diluted to 2 M urea, pooled into dialysis tubing (SnakeSkin^TM^, 10,000 MWCO, Thermo Scientific), and dialyzed in Tris-buffered saline (TBS) (6.05 g tris HCl, 9.0 g NaCl, 1 L of cold ddH_2_O, pH 7.4) containing 5 mL of chloroform at 4 °C for at least 4 h. Two more rounds of dialysis in TBS without chloroform at 4 °C overnight was followed. The ECM protein extract within the dialysis tubing was concentrated with polyethylene glycol (PEG) in 4 °C on a rocker until the solution reached a desired viscosity and transferred to a fresh tube for experiments or flash-freezing in liquid nitrogen for storage.

### 2.6. Measurement of Elastic Modulus by AFM

Force measurements of human breast tissue cross sections or HB-TMG were done using a Bruker (Dimension Icon ScanAsyst, Santa Barbara, CA, USA) atomic force microscope (AFM) with a 0.6 μm silicon conical tip on nitride lever (SNL-10-D Bruker) and a spring constant rating of 0.06 N/m to assess the elastic modulus of both the gel and tissue samples. In this work, the term elastic modulus is exclusively referred to the Young’s modulus. The tissue samples embedded in optimal cutting temperature (OCT) compound were cross sectioned (20 μm), mounted on silane-coated slides, rinsed with 1× PBS and ddH_2_O to remove OCT. The samples were kept at 4 °C until AFM measurement. Before AFM, the samples were allowed to warm up to RT in a sealed plastic bag to prevent drying. The ECM hydrogel coated on silane slides were kept in 1× PBS until measurement, at which time the PBS was decanted. The AFM probe tips were calibrated before each use. For calibration, three force–distance curves were taken using the hard calibration sample provided by the AFM manufacturer. From these curves, the deflection sensitivity was calculated and then averaged. Once the deflection sensitivity was updated, the thermal tune function was enabled to calibrate the spring constant. Each time the calibration indicated that the deflection sensitivity ranged from 92 nm/V to 112 nm/V. The spring constant, expected to be 0.06 N/m, ranged from 0.062 to 0.075 N/m. During the tip calibration process, the samples were removed from respective storage locations and allowed to acclimate to RT, and for the gel samples, excess water was carefully removed by pipette. Measurements were taken at the highest points available in the sample to avoid pores and pockets as much as possible. Gel samples were wetted intermittently using 1× PBS solution to maintain moisture levels for measurements. At least seven measuring spots on each sample were used for data acquisition. Ramp mode was used to collect indentation on the sample and generate a force curve reading. Force curves were then fit using the Hertz model and modulus values recorded.

### 2.7. IF Staining of Polymerized Hydrogel

The staining was performed as we previously reported [29] with modifications. Briefly, for staining of type I collagen (Col I) in HB-TMG, pig breast tissue matrix gel (PB-TMG; homemade as previously reported [28]), human Col I (Advanced BioMatrix, #5007), or Matrigel (Corning, #356230), silicone wells attached to Silane-Prep slides (Sigma) were coated with 10 µL of the hydrogels at the concentrations that will have elastic moduli similar to those of native tissue ECM upon polymerization as reported [29] or defined in the current study. The gels were polymerized in a 37 °C incubator for 1 h, followed by fixation with 4% paraformaldehyde, washing with 1× PBS, blocking with 3% BSA in PBS, incubation in Collagen I antibody (Novus Biologicals, Centennial, CO, USA; #NB600-408) at 4 °C overnight, and subsequent incubation in Alexa Fluor 488-conjugated secondary antibody (Thermo Fisher Scientific) at RT in dark for 1 h. After washing three times with 0.1% Triton-X 100, the silicone rings were removed, the gels were briefly air-dried, FluorSave mounting medium (MilliporeSigma, Burlington, MA, USA; #345789) was dropped at the top of the gels, and coverslips were mounted to the cover the samples. The slides were stored in dark at 4 °C overnight and imaged using an epifluorescence microscope.

### 2.8. Machine Learning of ECM Hydrogel Microstructures

A Convolutional Neural Networks (CNN) Autoencoder [31] was applied to analyze the IF images for distinct differences or similarities in the microstructures of four different types of native tissue ECM hydrogels, HB-TMG, PB-TMG, Col I, and Matrigel, with 12 individual IF images for each type of hydrogel (Figure S2). To build up the training dataset, the ROOSTER software (https://zzlab.net/Rooster, accessed on 14 October 2021) was used to divide each image into 120 tiles. In total, 5760 tiles were generated as a training dataset for the neural network. Each tile was a 3D structure with measurements of 104 × 120 × 3. Value 3 represents the three Red, Green, and Blue (RGB) bands. The Autoencoder architecture consisted of 9 layers. Layers 1 through 5 used the 2D convolution method and max-pooling method to encode main features from the input tiles. Layers 6 through 9 used the up-sampling method to decode features learned from the first five layers. Cross-entropy loss between the original and the predicted tiles’ pixels was applied to train the model. The fifth layer of the model output size was 13 × 15 × 16, which contained the abstract encoded features. The value 16 represented 16 types of learned features. The 2D matrix for each feature type was averaged and obtained a 1D array of 16 values to represent each tile. The ROOSTER software was used to remove tiles that contained no information or too many fibers. Principal Component Analysis (PCA) and feature plots (Figure S3) were applied to the rest tiles to examine if they were useful for separating the four types of IF images. The 16 values were also applied as the threshold to generate feature extraction masks. We used Keract [32] to produce outputs of the fifth layer of the Autoencoder for tiles, applied the averaged features to filter out pixels less than the threshold, and generated 16 feature masks for each tile (Figure S4).

### 2.9. Cell Culture

MDA-MB-231, MDA-MB-468, T47D, BT474, and SKBR3 breast cancer cells (ATCC) were grown in 1× DMEM containing 10% fetal bovine serum (FBS) and 1% penicillin and streptomycin. MCF10A human mammary epithelial cells (ATCC) were grown in DMEM/F12 contained 5% horse serum, 20 ng/mL EGF, 1% penicillin/streptomycin, 0.5 μg/mL hydrocortisone, 100 ng/mL cholera toxin, and 10 μg/mL human insulin. Normal primary human mammary epithelial cells (HUMEC, ScienCell Research Laboratories, Carlsbad, CA, USA) were grown in Mammary Epithelial Cell Medium (ScienCell Research Laboratories). All cells were cultured in a 37 °C incubator (supplied with 5% CO_2_).

### 2.10. Breast Epithelial Cell 3D Growth within Breast ECM Hydrogel

MCF10A cells were mixed with the breast ECM hydrogel at a density of 2000 cells/40 μL of gel and seeded in a well of an 8-well chamber slide. A volume of 400 μL of culture medium was added to the well. The cells in the gel suspension were cultured for up to 12 days with the addition of 17β-Estradiol (E2, final concentration 300 pg/mL) or/and insulin-like growth factor-1 (IGF-1, final concentration 300 ng/mL) every other day. 3D cell growth within the gels were imaged using phase contrast microscopy. The length and width of 15 cell-aggregates captured on three independent images for each experimental condition were measured based on the scales on the images. The sizes of the aggregates were calculated using the equation (length × width^2^)/2. The mean size of the aggregates of each experimental group was used for comparison analyses by one-way analysis of variance (ANOVA) and Post Hoc Tukey honestly significant difference (HSD). A *p* value of <0.05 was considered significantly different.

### 2.11. Breast Epithelial Cell Morphology on HB-TMG

The 8-well chamber slides were placed on ice and coated with 5 μL/well of human breast ECM hydrogel. The gel was polymerized in an incubator (37 °C, 5% CO_2_) for approximately an hour. A total of 2000 cells/well were resuspended in 400 μL culture medium, seeded on the polymerized hydrogel, and cultured for 24 h under optimal conditions. Then, the cells were fixed with 4% paraformaldehyde, permeabilized with 0.1% Triton-X 100 in PBS, blocked with 3% BSA, stained with Alexa Fluor probe-conjugated phalloidin (Thermo Fisher Scientific, #A22284) and Hoechst (TOCRIS, #5117) as reported [29]. Col I primary and fluorophore-conjugated secondary antibody probing for Col I in the hydrogel was performed as described before. IF imaging of the stained cells that were embedded in HB-TMG and the cells cultured on 2D glass surface (prepared parallelly for the experiment) was done over a fluorescence microscope. Four to seventeen representative cells from each cell type were selected for quantitative analysis. The mean numbers of the protrusions on the cells cultured under 2D or 3D conditions were used for comparison analyses by one-way analysis of variance (ANOVA) and Post Hoc Tukey honestly significant difference (HSD). A *p* value of <0.05 was considered significantly different.

### 2.12. Acini Formation

A volume of 40 μL/well of HB-TMG was added into the wells of 8-chamber slides and polymerized in a 37 °C incubator (supplied with 5% CO_2_) for approximately 1 h. A total of 4000 cells/well were resuspended in 400 μL of culture medium and cultured for 7–10 days. The cells were fixed with paraformaldehyde, permeabilized, and incubated with primary antibodies against E-cadherin (Thermo Fisher Scientific, #701134) and β4 integrin (R&D Systems, Minneapolis, MN, USA; #MAB4060), followed by fluorophore-conjugated secondary antibodies and Hoechst staining as described above, imaged for acinar structures under a fluorescent microscope.

## 3. Results

### 3.1. Human Normal Breast and Invasive Breast Cancer ECM Structures and Mechanical Properties

**Figure 1:**
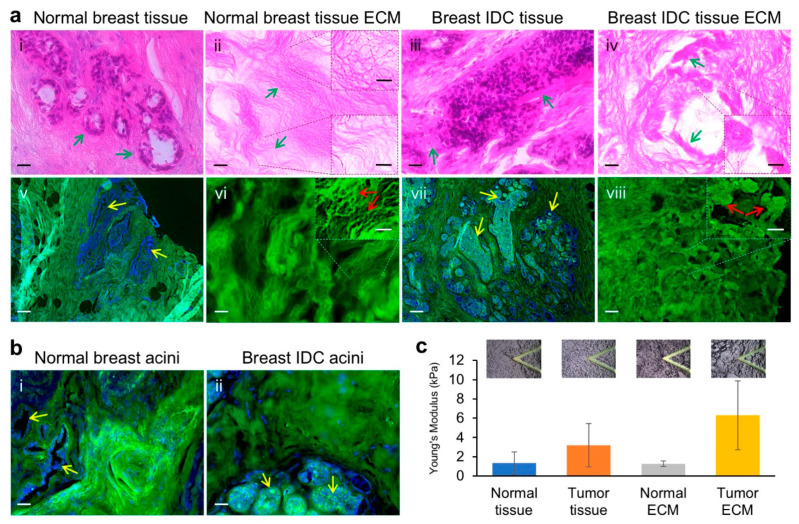
Histopathological features of normal and invasive ductal carcinoma tissues and ECM. (**a**) H&E staining (upper panels) of the cross sections of the normal breast tissue (**i**), decellularized normal breast tissue ECM (**ii**), breast IDC tissue (**iii**), and decellularized breast IDC tissue ECM (**iv**) as well as IF staining (bottom panels) of the corresponding tissue or ECM sections (**v**–**viii**). Green stain, Col I; blue stain (Hoechst), nuclei; white stain, Ki-67. Arrows indicate the normal or cancerous acini or ECM structures. Insets magnify the indicated regions of interest. Scale bars: (**i**–**iv**,**vi**,**viii**), 20 μm; (**ii**,**iv**) insets, 10 μm; (**v**,**vii**), 100 μm; (**vi**,**viii**) insets, 50 μm. (**b**) High magnification of IF images showing the acinar structures void of Col I staining of the breast epithelial cells (yellow arrows) in normal breast tissues (**i**) and positive for the infiltrated cancer cells (yellow arrows) within the acini lumens in IDC tissues (**ii**). Green stain, Col I; blue stain (Hoechst), nuclei; white stain, Ki-67. Scale bars: (**i**,**ii**), 20 μm. (**c**) Graphed Young’s modulus values for the elasticities of the tissues or ECM measured with AFM. A minimum of seven measuring spots on three consecutive cross sections of each sample were used for data acquisition. Representative microscopic images of tip-probing status were exhibited on top of the values of the individual samples. Error bars, standard deviation (SD).

To better understand the destructive features of breast cancer tissues, we carried out hematoxylin and eosin (H&E) as well as type I collagen (Col I) immunofluorescence (IF) staining on the cross sections of invasive ductal carcinoma (IDC) tissue, normal breast tissue, and the extracted ECM of the two types of tissues. The H&E staining results showed that while the normal mammary gland acinar structures remained intact, with epithelial and myoepithelial cells lining along the acini walls (Figure 1a(i)), the IDC lesion acinar structures were destroyed, with unclear lumen boundaries and disorganized abundant cells (Figure 1a(iii)). Additionally, compared to the normal acini surrounding connective tissue structures, which had eosin staining at a density and pattern like that of the tissues around it, the tissue structures next to the cell-rich lesion region appeared to be denser and stained by eosin as a much darker pink color compared to the surrounding connective tissues (Figure 1a(i,iii), green arrows). Examination of the H&E staining of the normal breast tissue ECM showed an overall even staining of the ECM textures (Figure 1a(ii)). Two major structural features on the ECM were clearly recognizable: a mesh-like structure and a long fibrous structure (Figure 1a(ii), insets). In contrast, the staining pattern on the IDC tissue ECM sections were non-even, with thick ECM aggregates, which likely corresponded to the dark and dense eosin staining of the tumor surrounding tissue areas in the IDC tissue sections (Figure 1a(iv,iii)). These normal breast ECM and tumor tissue ECM characteristics were more clearly seen in high magnification images (Figure S1).

Similar to the H&E staining data, IF staining of the tissue sections showed that the normal mammary gland acinar structures were well organized within the collagen-rich connective tissues as highlighted by the nuclei Hoechst staining of the acini cells and tissue Collagen I (Col I) staining (Figure 1a(v)), whereas cancer cell occupancies within the acini lumens at different levels and deformation of the regular acini to enlarged and irregular structures were clearly observed (Figure 1a(vii)). Staining of Col I within the decellularized breast tissue ECM demonstrated mesh-like and fibrous structures in the normal ECM and aggregated ECM in the IDC tissue ECM (Figure 1a(vi,viii)), similar to those seen in the ECM H&E Staining. Interestingly, while the luminal epithelial cells of the normal breast tissues were absent of Col I staining, many of those lining the inside wall of and accumulated within the acini lumens of the IDC tissues were strongly stained for Col I (Figure 1a(v,vii),1b(i,ii)).

To examine the mechanical properties of the breast tissues and decellularized tissue ECM, atomic force microscopy (AFM) was performed following our established protocol [28] with modifications for human tissues. Young’s moduli of the samples were: normal breast tissue 1.328 ± 1.174 kPa; IDC tumor tissue 3.193 ± 2.256 kPa; normal breast tissue ECM 1.263 ± 0.294 kPa; and IDC tumor tissue ECM 7.203 ± 3.255 kPa. It appears that the tissue heterogeneity had made the standard deviations of the values of the measurement-points larger than those normally seen in measuring mechanically even surfaces. Clearly, the tumor tissues were approximately 2.4-fold stiffer than the normal tissues. The tumor tissue ECM was approximately 5.7-fold stiffer than the normal tissue ECM. Interestingly, while the normal breast tissue and its ECM had similar elastic moduli, the IDC tumor tissue ECM was approximately 2.3-fold stiffer than the IDC tumor tissues, suggesting changes in the ECM mechanical forces.

### 3.2. Human Breast ECM Protein Hydrogel Generation and Microstructural Features

**Figure 2:**
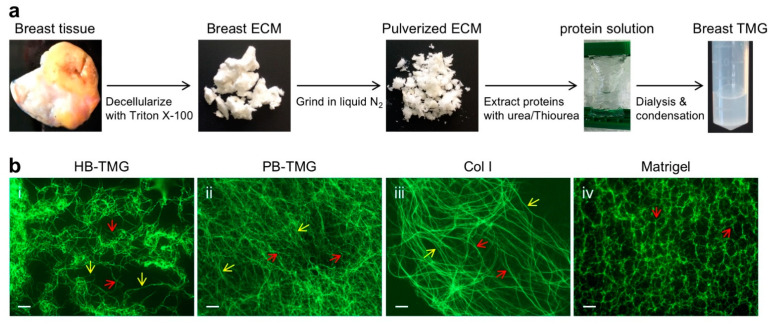
HB-TMG generation and microstructural differences from other ECM-derived hydrogels. (**a**) Flowchart for normal breast tissue ECM extraction and HB-TMG production. (**b**) Microscopic IF images of HB-TMG (**i**), PB-TMG (**ii**), Col I (**iii**), and Matrigel (**iv**) polymerized at the elastic moduli similar to native tissues. Red arrows, collagen fibers with connections or branches; yellow arrows, long collagen fibers. Scale bars, 10 μm.

Our previous work has established protocols for mouse and pig breast tissue ECM extraction and hydrogel generation [11,28]. In the current study, we adapted the pig breast ECM whole protein extraction method [28] with optimized modifications for human breast tissues as detailed in the methods. Normal breast tissues spared from contralateral prophylactic mastectomy after surgical treatment and diagnostic needs were subjected to decellularization with detergent to obtain tissue ECM, followed by grinding the ECM in liquid nitrogen and extracting ECM proteins with a series of urea or/and thiourea buffers at an increasing gradient (Figure 2a). This method ensures the extraction of the majority of ECM proteins at various native states and with different solubilities. The pooled extracts were adjusted to a final urea concentration of 2 M, dialyzed extensively, and condensed to a concentrated hydrogel stocking solution that was two-fold thicker than a final working solution. The ECM hydrogel working concentration was defined by AFM measurement of the Young’s moduli of polymerized hydrogels, which had a similar stiffness as that of the extracted ECM (1.263 kPa) derived from normal breast tissues (Figure 1c), following the procedures we previously reported [28]. The Young’s modulus for the hydrogel used in the experiments of this work was 1.260 kPa. Culture medium was used to dilute the stock hydrogel solution. To be consistent with the hydrogel terminology used in our previous work [29], we termed the hydrogel Human Breast Tissue Matrix Gel (HB-TMG), whereas the hydrogel derived from porcine breast tissues, which was also used in this study, was acronymed PB-TMG.

To examine the microstructures of the polymerized HB-TMG associated with certain cellular phenotypes in 3D tissue cultures, we coated the hydrogel on glass slides, polymerized in an incubator at 37 °C (5% CO_2_), stained with antibody for Col I that is a major structural component of animal and human breast tissue ECM [11,28,33,34] (Figure 1a(ii,vi)), and imaged under a fluorescence microscope. Our results showed that the polymerized collagen fibers in HB-TMG were interconnected long strands, forming an intricate fibrous network (Figure 2b(i)) in a pattern resembling that of decellularized breast tissue ECM (Figure 1a(ii,vi)). This complex structural feature of the HB-TMG matrix was like that observed in polymerized PB-TMG (Figure 2b(ii)) and distinct from the polymerized collagen I, which displayed very long fibrous and stranded structures with minimal crosslinks or interconnections between the fibers (Figure 2b(iii)). In contrast, the collagen fibers in polymerized Matrigel were short with different thickness and interconnected, forming distinguishable honeycomb-like structures (Figure 2b(iv)) as we previously reported [29]. These data collectively indicate that different tissue ECM sources can give rise to ECM hydrogels with quite different microstructures.

### 3.3. Comparing the Microstructures of Human Breast ECM Protein Hydrogel and Other Native Tissue ECM Hydrogels Using Machine Learning

Machine learning technology is very useful for transferring visualized features to numerical values. An investigation was conducted using the technology to represent the visual differences between the human breast ECM hydrogel and the other native ECM hydrogels by numerical values. Specifically, the distinct or the similar features of the collagen fiber structures captured on the different types of hydrogel images (Figure 2b). Twelve different IF images (data not shown) for each of the HB-TMG, PB-TMG, Col I, and Matrigel groups were selected for the analysis. A Convolutional Neural Networks (CNN) supercomputing program, Autoencoder [31], was used to analyze the IF images (Figure S2).

**Figure 3:**
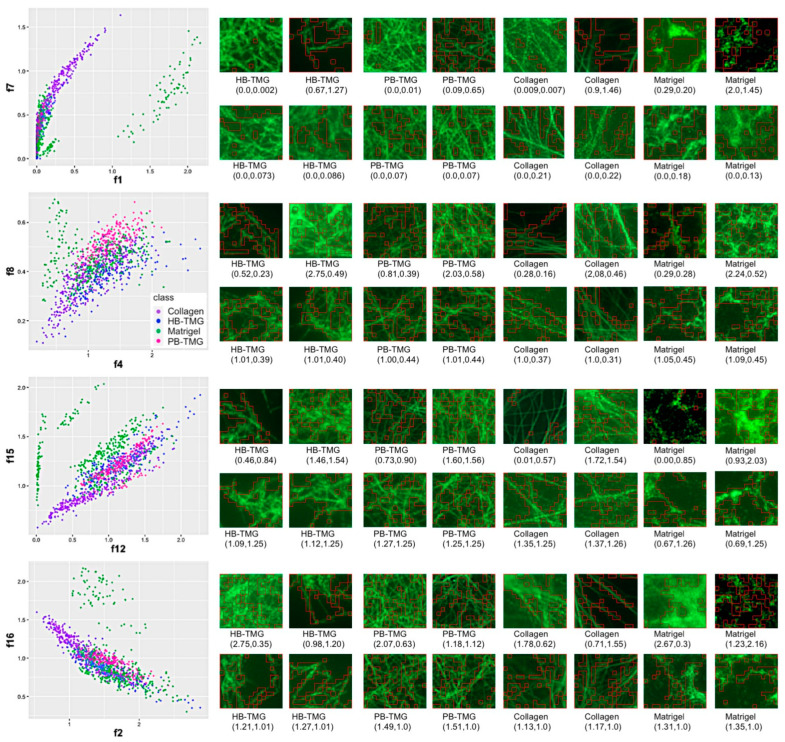
Machine learning analysis of microstructural distinctions between the different native ECM hydrogels. Four groups of figures are presented. Each group contains a scatter plot of learned features and 16 tiles with feature highlights of four kinds of IF staining images. The top eight images represent the features of maximum and minimum values selected from the left plot. Additionally, the bottom images represent features of similar values. Values under each image are the corresponding values in the left figure.

Eight key features were extracted from the pooled feature analyses (Figure S3) to represent the features learned from Autoencoder. Features 1, 8, 15, and 16 were sufficient to separate the four types of IF images. Features 2, 4, 7, and 12 illustrated what was learned. Four groups of plots and tiles with highlighted feature areas were extracted from the analysis (Figure 3). Each group consisted of a feature plot (Figure 3, left) and 16 tiles with highlights of learned features (Figure 3, right). Highlighted areas are generated from features’ masks (Figure S4). The corresponding feature values under each title indicated the sampled tile’s position from the corresponding plot figure. The top eight tiles were selected from the maximum and minimum feature values for distinct structural features on the image. The bottom eight tiles were chosen from the areas where most tiles share similar feature values for structural similarities on the images. The plot of feature 1 vs. 7 showed feature 1 separated the images into two parts; highlighted areas on the tiles to the right showed feature 7 extracted the hollow type of features. The plot of feature 4 vs. 8 showed feature 8 clustered the image tiles; tiles on the right showed feature 4 represented long fiber features from the image tiles. The plot of feature 12 vs. 15 showed feature 15 clustered the images well; tiles on the right showed feature 12 highlighted intersect features on the tiles. The plot of feature 2 vs. 16 showed that feature 16 clustered the images into two parts; feature 12 also highlighted intersect features on the images.

These analyses defined the major differences and similarities of the microstructural features among the four different types of hydrogels and highlighted the key features of interest from the pooled datasets for further analysis. Collectively, our data indicated that there were more structural similarities between HB-TMG and PB-TMG, which, in turn, have certain degrees of similarities with Col I structures, especially the fibrous structural features characteristic of fibril-forming collagens within human tissues [35]. Except for the meshwork-like features in Matrigel microstructures that resembles the interconnection within the HB-TMG and PB-TMG matrices, the majority of Matrigel structural features are distinct from those of the other three matrices.

### 3.4. 3D Growth of Human Mammary Epithelial Cells in Human Breast Tissue ECM Hydrogel

**Figure 4:**
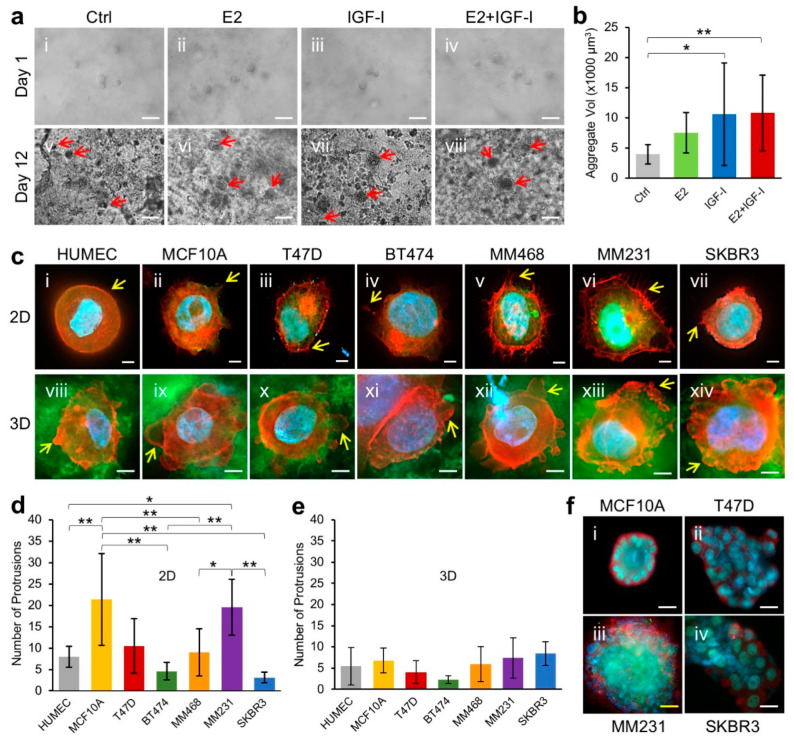
HB-TMG support of human breast epithelial cell 3D growth. (**a**) MCF10A cell suspending growth within HB-TMG in the presence or absence of E2 or/and IGF-I stimulation. Arrows, cell spheres. Scale bars, 50 μm. (**b**) Quantification of the volumes (vol) of the Day 12 MCF10A cell 3D aggregates shown in (**a**). * *p* < 0.05; ** *p* < 0.01. (**c**) IF staining of normal breast or breast cancer epithelial cell culture on 2D surfaces or 3D HB-TMG. Arrows, cell protrusions. Red, phalloidin; green, Col I; blue, nucleus Hoechst staining. Scale bars, 5 μm. (**d**) Quantification of cell protrusions in 2D cultures. * *p* < 0.05; ** *p* < 0.01. (**e**) Quantification of cell protrusions in 3D cultures. (**f**) IF staining of acini formation of normal breast or breast cancer epithelial cells. Red, β4 integrin; green, E-cadherin; blue, nucleus Hoechst staining. Scale bars: c-i, c-ii, and c-iv, 20 μm; c-iii, 50 μm.

Female mammary epithelium growth and breast development require coordinated stimulations by the estrogen steroid hormone 17β-Estradiol (E2) and growth factors, such as insulin-like growth factor-I (IGF-I) [36,37,38]. To recapitulate the stimulatory effect of E2 and IGF-I on breast epithelial cell growth in a human native breast tissue-like microenvironment, we carried out a cell-suspension 3D growth experiment using the human breast ECM hydrogel as a culture substratum. Briefly, MCF10A human normal mammary epithelial cells were suspended in HB-TMG, which was prepared having an Young’s modulus like that of human normal breast tissue ECM (Figure 1c), and cultured on 8-well chamber slides for 12 days. E2 or/and IGF-1 was added to the cultures at a final concentration the same as that in the circulation of normal female in their 20s every other day [39,40,41,42,43]. The 3D growth of the cells was monitored and imaged under a light microscope. The results showed that while the cells in the E2, IGF-I, and E2 plus IGF-I exhibited single-cell suspension states like those seen in the control samples on the Day 1 of the cell culture (Figure 4a(i–iv)), spherical cell aggregates were formed in the cultures on Day 12. The aggregates in the E2-treated cultures were 1.92-fold larger than the control cultures, and those in the IGF-I-treated or the E2 plus IGF-I-treated cultures were even larger than the E2-treated cultures and were 2.68-fold and 2.74-fold, respectively, larger than the control cultures. (Figure 4a(v,viii),b).

Next, we inspected the morphologies of normal breast epithelial cells and breast cancer cells representing the major molecular types of breast cancers grown on tissue culture-grade 2D glass slide or 3D HB-TMG surfaces for 24 h. The cells used in the experiments were primary normal human mammary epithelial cells (HUMEC), MCF10 immortalized normal human mammary epithelial cells, T47D cancer cells (Luminal A type; ER^+^, PR^+/−^, HER2^−^), BT474 cancer cells (Luminal B type; ER^+^, PR^+/−^, HER2^+^), MDA-MB-468 cancer cells (MM468, Basal A type; ER^−^, PR^−^, HER2^−^), MDA-MB-231 cancer cells (MM231, Basal B type; ER^−^, PR^−^, HER2^−^, claudin-low), and SKBR3 cancer cells (HER2-overexpressing). An observed morphological feature distinct between the 2D and 3D cultures was that both normal and cancer cells grown on the 2D surface displayed spike-like or needle-like filopodia protrusions [44], whereas those cultured on the 3D HB-TMG exhibited rounded blebbing or pseudopod-like protrusions [44,45] (Figure 4c). Quantifications of the protrusions indicated that MCF10A and MM231 cells had significantly more protrusions than HUMEC, BT474, and SKBR3 cells while T47D and MM468 cells displayed insignificantly more protrusions than HUMEC, BT474, and SKBR3 cells in the 2D cultures (Figure 4d). Though MCF10A, MM468, MM231, and SKBR3 cells exhibited more protrusions than HUMEC, T47D, and BT474 cells in the 3D cultures, the differences between their protrusion numbers were insignificant (Figure 4e).

We previously reported that normal mammary epithelial cells formed acinar structures when cultured on porcine breast tissue ECM protein hydrogel PB-TMG [28,29]. Here, we assessed the 3D acini formation capacity of both normal and cancerous mammary epithelial cells on human normal breast tissue ECM protein hydrogel HB-TMG. MCF10A cells or T47D, MM231, and SKBR3 cancer cells, which represent each molecular type of breast cancers were grown on a thick layer of HB-TMG coated at the bottom of the wells of 8-well chamber slides and cultured for up to 10 days, followed by IF staining and imaging analysis as specified in the method. Our data showed that MCF10A cells formed regular acinar structures on the matrix. The cells along the edge of a well-developed acini had organized cell–cell contacts, with β4 integrin (cell membrane receptor for laminins) highly expressed on the cell surfaces that directly contacting the hydrogel matrix (Figure 4f(i)). In contrast, the cancer cells formed irregular aggregates without lumen-like structures, even if the aggregates became very large over time as in the case of the MM231 cell cultures (Figure 4f(ii,iv)). MM231 cell aggregates were overall much larger than the MCF10A acini and the aggregates formed by T47D or SKBR3 cells within the culturing period. Since many big MM231 cell aggregates were out of frame for imaging under the same magnification as used for other cell aggregates and some of the T47D cell or SKBR cell aggregates were in irregular shapes, we did not quantify the sizes of the aggregates. Instead, the phenotypic data were exhibited. Though β4 integrin expression was detectable in the cancer cells, the localization of the integrin receptors at the surfaces of the cancer cells were low, and there was no clear accumulation of the receptors along the edges of the cancer cell aggregates (Figure 4f(ii,iv)). These data collectively suggest that HB-TMG supports both normal and cancerous mammary epithelial cell 3D growth and interaction with their ECM microenvironments at both physical and membrane receptor-ECM ligand coupling levels.

## 4. Discussion

A couple of novel insights into human breast tissue microenvironment and mammary epithelial cell biology within the environment have been revealed through this study. First, human normal breast and invasive breast cancer tissue ECM structures devoid of tissue cells and non-structural tissue components were characterized, exhibiting a clear structural definition of the ECM beyond those observed at gross tissue levels and providing references for in vitro close mimicry of human breast tissue matrices. Second, generating human breast tissue ECM protein hydrogel has made culturing and examining human mammary cells in their native microenvironment possible. Third, defining the microstructural features of the human breast ECM hydrogel, HB-TMG, and comparing them with those of other non-human breast tissue-derived native hydrogels using supercomputing machine learning technology have uncovered the structural complexities and distinctions of the different native ECM, implicating the importance of tissue-specific microenvironment-mimicry in biological studies of specific tissue cells. Fourth, inspecting the 3D growth of human normal or cancerous mammary epithelial cells within their native ECM microenvironment is a closest in vivo-mimicking culture condition for cell biology studies. We expect these findings will facilitate breast cancer modeling and research.

Early work in animal tumor models has identified tumor-associated collagen signatures (TACS) around mammary tumors [46]. The three canonical TACS range from TACS-1, which is defined by increased collagen density near a tumor, to TACS-2 that describes stretched collagen fibers around a tumor and TACS-3 that designates irregular or perpendicular collagen fiber alignment to a tumor. TAC-3 has been found to be related to invasive breast lesion types and worse prognosis compared to those lesions without the TAC-3 characteristics [47]. In the past 15 years, clinical evidence has confirmed the TACS [47,48,49,50]. Our decellularized normal breast ECM and breast tumor ECM imaging analyses not only clearly confirmed all the TACS phenotypes described above (Figure 1a(iv) vs. Figure 1a(ii)), but also introduced additional cancer-associated ECM features, at least for the IDC type of tumors. These pathological ECM features are collectively exhibited as: (1) thickening of ECM around the lesion (Figure 1a(iii)); (2) broken fibrous ECM textures (Figure 1a(iv)); (3) collagen aggregation in or around the lesion region (Figure 1a(iv,viii)); (4) collagen particle accumulation within the affected acini lumens along with infiltrated cancer cells (Figure 1a(vii),b(ii)). Whether stromal cells, such as fibroblasts, also infiltrated into the luminal structures and contributed to the luminal collagen accumulation requires further investigation. Interestingly, while it is believed that breast tissue fibroblasts are the chief stewards for collagen deposition in breast tissues [51], it was reported that tumor/cancer-associated fibroblasts (TAF/CAF) in invasive breast cancer had attenuated generation of collagen [52], which was, instead, produced by cancer cells [53,54]. These observations seem to be consistent with the luminal infiltrated cancer cells and accumulated collagen phenotypes identified in this study.

To define the biological activities of human breast epithelial cells within their native microenvironment, we extracted whole proteins from normal human breast tissue ECM and generated a hydrogel, HB-TMG, for cell cultures. When polymerized at body temperature, the collagen within HB-TMG forms a meshwork structure (Figure 2b(i)), with interconnected fibers, like that seen in the decellularized breast tissues (Figure 1a(ii,vi)). The advantages of using the native breast ECM protein hydrogel as cell culture substratum are multifold: (1) cells grown in the hydrogel with native tissue ECM elasticity are within a 3D suspending environment—a condition similar to that of their native living tissues; (2) the hydrogel contains all the ECM structural proteins, which serve not only as structural support for the cells living in the matrix; (3) the ECM proteins, such as Col I, Col IV, laminin, and fibronectin, are themselves extracellular signaling ligands for cell membrane receptors, such as integrins [9,55,56]. Activation of intracellular signaling cues via the ECM protein-membrane receptor coupling is necessary for cellular biological functions, especially for the adaptation and response of the cells to the changes within the ECM microenvironment; (4) The transparent nature of the gel enables imaging observations of the morphologies and behaviors of the cells grown in it. Thus, HB-TMG provides a tissue-specific native microenvironment for the tissue-specific cells, reconstituting an ideal model for the discovery of novel aspects of native cell biology.

Comparison of the microstructures of HB-TMG with PB-TMG, Col I, and Matrigel using IF imaging coupled with machine learning delineated the similarities and differences between the physical properties of the native matrices. These data are informative for cell biology studies using different biological matrices for 3D cell cultures since the extracellular signal-sensitive tissue-specific cells may only exhibit certain tissue-specific phenotypes or biological activities within the compositionally and structurally defined ECM microenvironment resembling that of the native tissues they live in. Additionally, our study has demonstrated the great power of machine learning in differentiating subtle differences between the microstructures of the matrices. This method could be very useful for further studies analyzing breast epithelial cell spatial phenotypes, such as sequential migration or invasion events, within the 3D networks of the ECM matrices. Additionally, the machine learning-aided ECM analytical model has great potential for novel discoveries of pathological changes in patient specimens that warrants further investigations.

While estrogen and growth factors are essential for women during normal breast development, abnormal E2 fluctuations during a woman’s lifetime and increased circulating IGF-I are associated with breast cancer risks [57,58]. It was shown that E2 was able to induce normal mammary epithelial cell transformation in vitro [59]. Clearly, E2 and IGF-I have a growth-stimulating effect under both physiological and pathological conditions depending on the tipping of the homeostasis of the tissue microenvironment and the genetic status of the mammary epithelial cells. In this study, we mimicked human native breast tissue 3D microenvironment with HB-TMG for normal breast epithelial cell MCF10A cell growth under the stimulation of E2 and IGF-I. It turned out that the cells formed a spherical structure within the culturing timeframe but exhibited a slow growing process in the absence of the hormone or growth factor. In contrast, hormonal and growth factor stimulation augmented the spheroid growth, suggesting a close mimicry of stimulated mammary epithelium growth in human breast tissues.

In pursuit of distinguishing the phenotypes of normal and malignant human mammary epithelial cells in HB-TMG, we performed short-term and long-term 3D cell cultures. During a 24 h culture window, in addition to the distinct protrusion styles on the cells grown on HB-TMG vs. on 2D glass surface (Figure 4c), the bleb-like protrusions on the cells cultured on HB-TMG reflect the barrier-effect of the matrix around the cells that limits cell migration, under which situation the cells may tend to ‘squeeze’ through the porous structures within the matrix [45]. This phenotype indicates that a low attachment surface was encountered by the cells and lower adhesion, lower traction force, and lower motility modes compared to those aspects seen on 2D surfaces were adopted by the cells, which may use an amoeboid migration strategy to get around the barriers within an ECM structure and resume their elongated shape for mesenchymal migration when entering a less confined tissue space [60].

When the breast epithelial cells were allowed to grow for extended times on HB-TMG, normal breast epithelial cells formed acinar structures while the cancer cells formed irregular aggregates of varying sizes. The poor acini-formation capacity of the cancer cells is consistent with the nature and aggressiveness of the cancer cells, which have disrupted apicobasal polarity that is essential for a rotational movement of acini and the integrity of the acini architectures [61,62]. It is known that a laminin- and Col IV-rich basement membrane (BM) or a basement membrane-resembling hydrogel substratum, such as Matrigel, is necessary for normal mammary epithelial cells to form correctly polarized acinar structures in cultures [63], and Col I hydrogel alone does not support mammary epithelial cells to form properly polarized acinar structures [63]. However, both human breast ECM and porcine breast ECM are Col I-rich matrices [28], which contain minimum amount of laminin and Col IV, and yet their hydrogels are able to induce normal mammary epithelial cell acini formation and expression of laminin receptor β4 integrin on the surfaces of the cells contacting the ECM hydrogels, an indication of correct polarity of the acini. This phenotype of Col I-rich ECM hydrogel-induction of acini formation suggests a couple of possible mechanisms: (1) the epithelial cells produce or recruit laminin or other components within the matrix for acini formation; (2) Col I and other components within the breast ECM hydrogels, including the small amount of laminin and Col IV, have a combined capacity to support acini formation; (3) the minimum amounts of laminin and Col IV in the hydrogel are sufficient for acini formation; (4) other ECM components other than laminin and Col IV within the hydrogel are able to induce acini formation; (5) a general ECM hydrogel providing a cell-suspension condition would induce 3D cell proliferation to form acini. These possibilities are worth investigating for our better understanding of human breast structural development and for improved modeling of breast cancer initiation and development. It is interesting that the MCF10A cell-formed acini and MM231 cell-formed aggregates closely resemble the normal and IDC destructed mammary gland structures, respectively, in the human breast tissues shown in Figure 1. These collectively suggest that HB-TMG indeed can provide a primary breast tissue-like microenvironment for the breast epithelial cells to display their native phenotypes.

## 5. Conclusions

This study provides novel insights into human breast tissue ECM structural features at both microscopic and supercomputing levels. These features may serve as references for biomedical research of human breast cancer and for bioengineering modeling of the development and progression of the disease. Human breast tissue ECM-derived hydrogel represents a powerful tool for cancer cell biological studies and for future individualized medicine and pharmaceutical applications.

## Data Availability

The data presented in this study are available on request from the corresponding author.

## Supplementary Materials

The following are available online at https://www.mdpi.com/article/10.3390/cancers13225857/s1, Figure S1: Human breast tissue ECM microscopic structures; Figure S2: Workflow of Autoencoder analysis of microstructures on hydrogel IF images; Figure S3: Summary of defined key features and PCA; Figure S4: Generation of feature masks for representation of learned microstructural features of the hydrogels.
