# Supplementary material for: Human Breast Extracellular Matrix Microstructures and Protein Hydrogel 3D Cultures of Mammary Epithelial Cells
Source: Cancers (Basel). 2021 Nov 22;13(22):5857. doi: 10.3390/cancers13225857 (PMC8616054; doi:10.3390/cancers13225857)
Supplement: Supplementary file 1 [file cancers-13-05857-s001.zip › cancers-1444653-supplementary.pdf]

Supplementary Materials

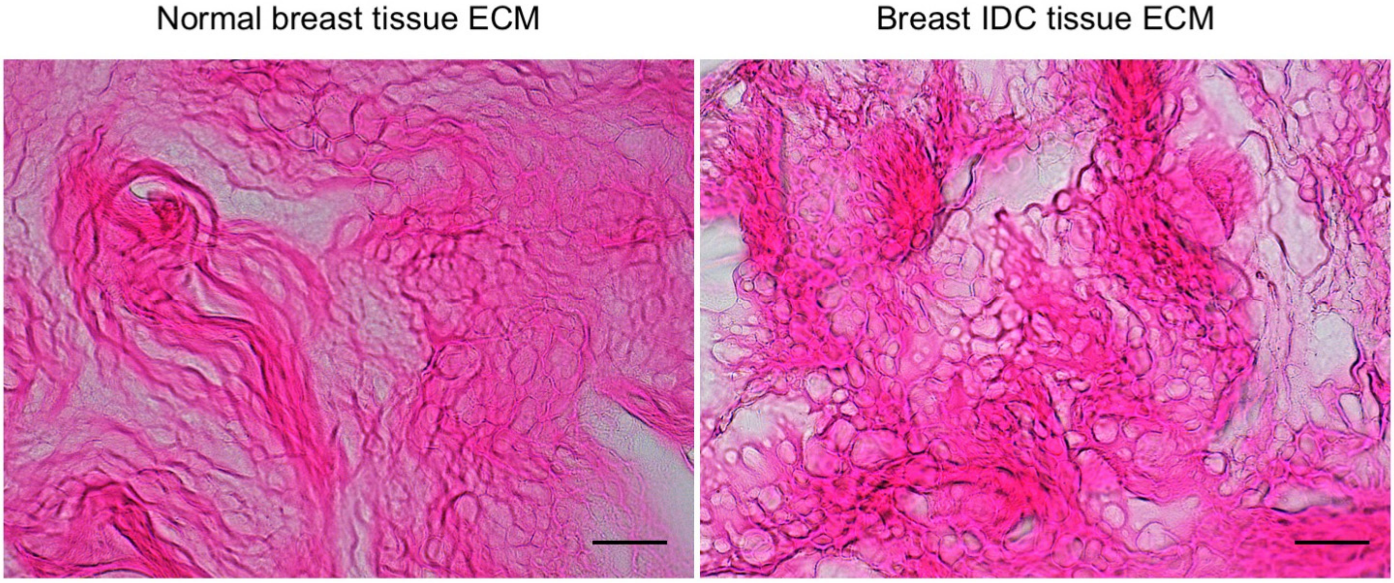

Figure S1: Human breast tissue ECM microscopic structures. Normal breast and IDC breast tumor tissue ECM cross sections from FFPE samples were stained with hematoxylin and eosin (H&E) and imaged under a high magnification light microscope. The mesh-like and the fibrous structures of the ECM were clearly visible in the normal ECM, while spread ECM aggregates at different sizes and thicknesses were identified in the IDC tissue ECM. Scale bars, 20 μm.

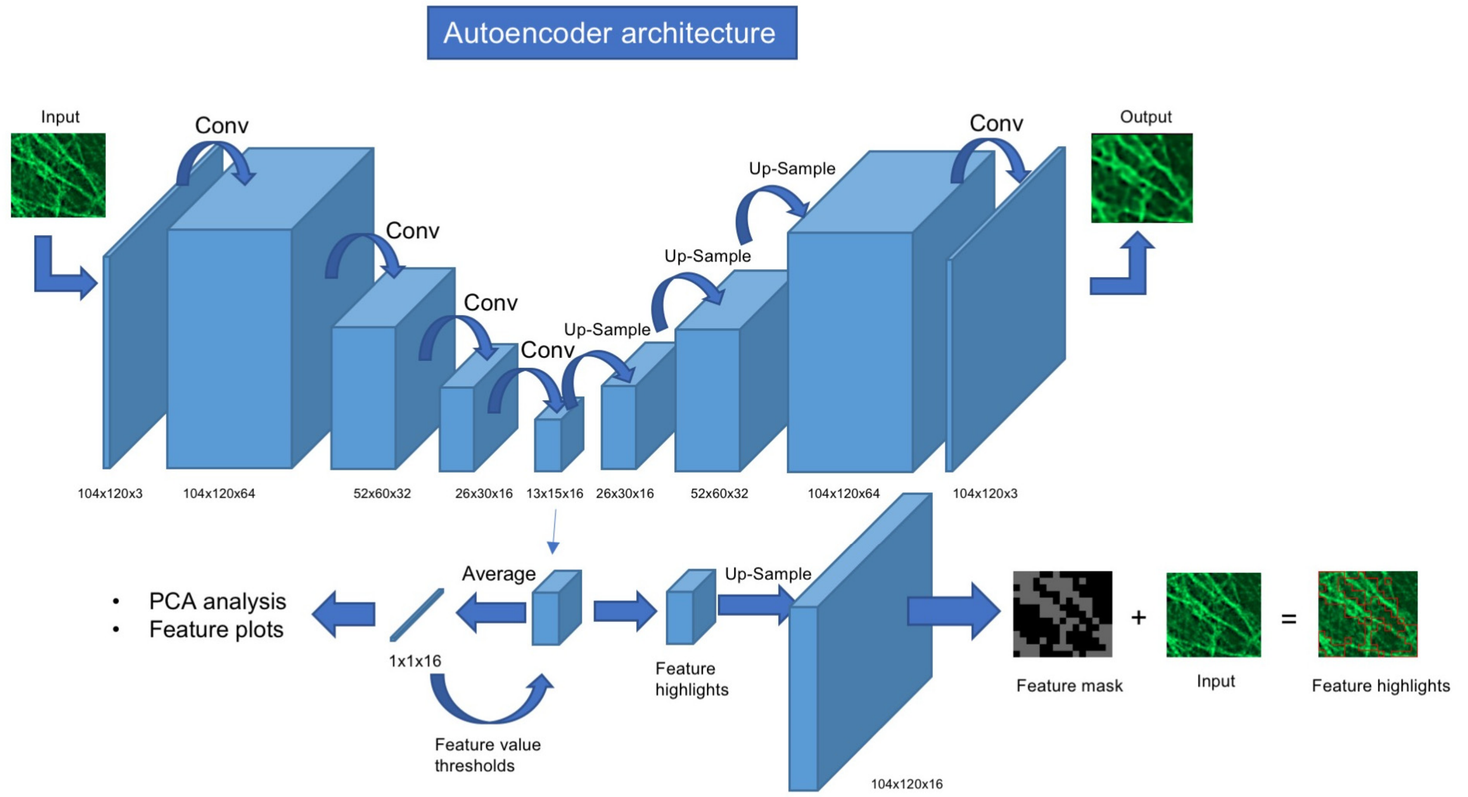

Figure S2: Workflow of Autoencoder analysis of microstructures on hydrogel IF images. An individual IF staining image was equally divided into 120 tiles using ROOSTER software. The input tile in the figure was an example of the split tiles. A Convolutional Neural Networks (CNN), Autoencoder[28], containing nine analytical layers, was built and trained. Each layer has three dimensions. A 2D convolution method was applied to learn image features. The training process aimed to find the best feature learner that was able to represent the tiles. The output image was generated by the Autoencoder, which mimicked the input image. The fifth layer (13×15×16) was the densest layer, which contained 16 feature learners for the abstract information features. The 2D values of each learner were averaged to become a 1D array with 16 values. These values were applied in two ways: feature exploration and feature mask generation. Feature exploration was performed by Principal component analysis (PCA) and scattered plots (Figure S3). Feature masks were then generated by pixels greater than the 16 values representing learned features (Figure S4). The lighter area in the feature mask represents the main features learned by the layer. Input image combined with the feature mask shows the contours of the highlight feature area.

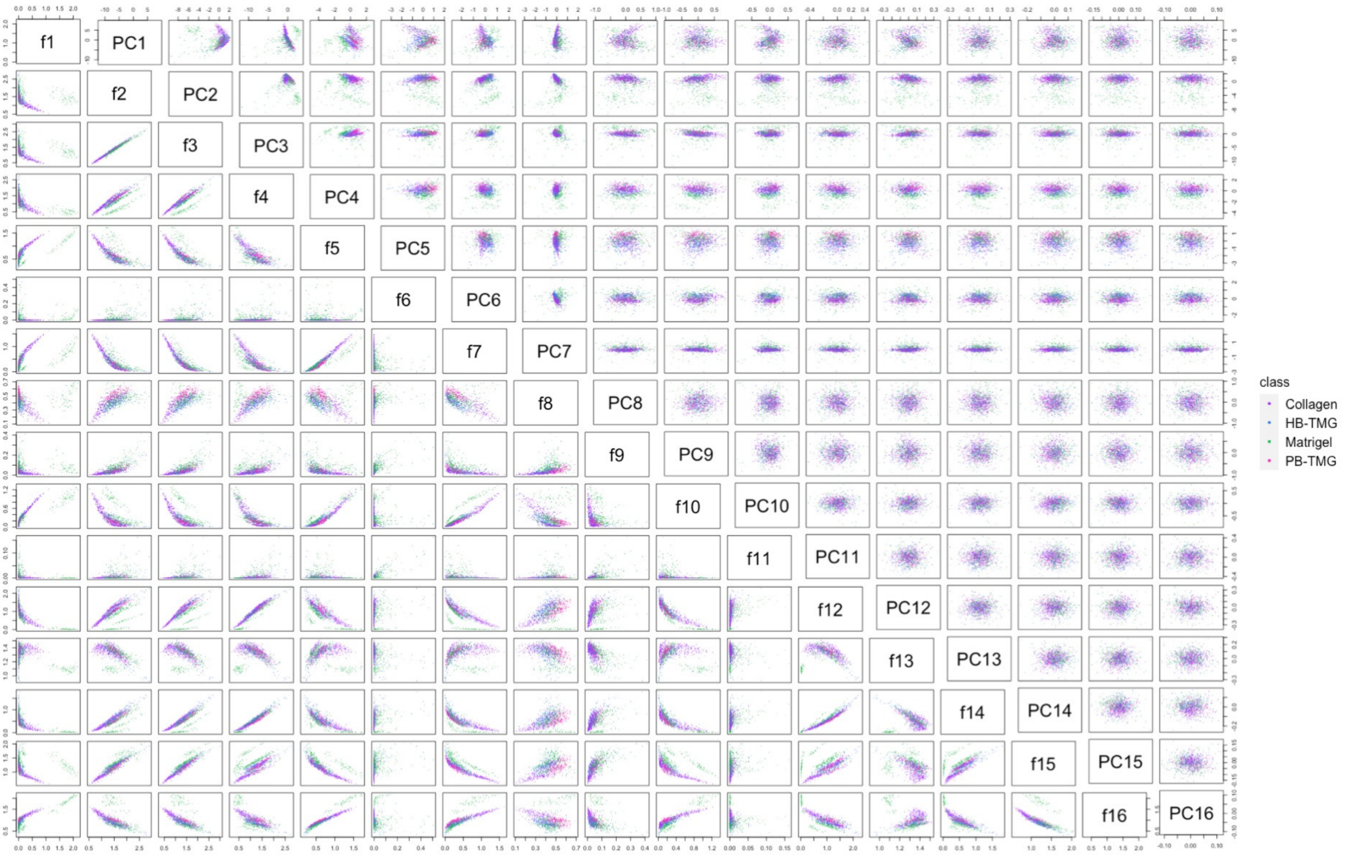

Figure S3: Summary of defined key features and PCA. The figure contains two sets of scatter plots. The bottom left represents pair-wise plots of 16 features extracted from each tile. The top right includes pair-wise scatter plots of principal components (PCs) from PCA of the 16 features. Features plots better separated the microstructural differences on the four groups of IF images than the PCs plots. Features that identify the biggest differences on the groups of images are f1, f8, f15, and f16.

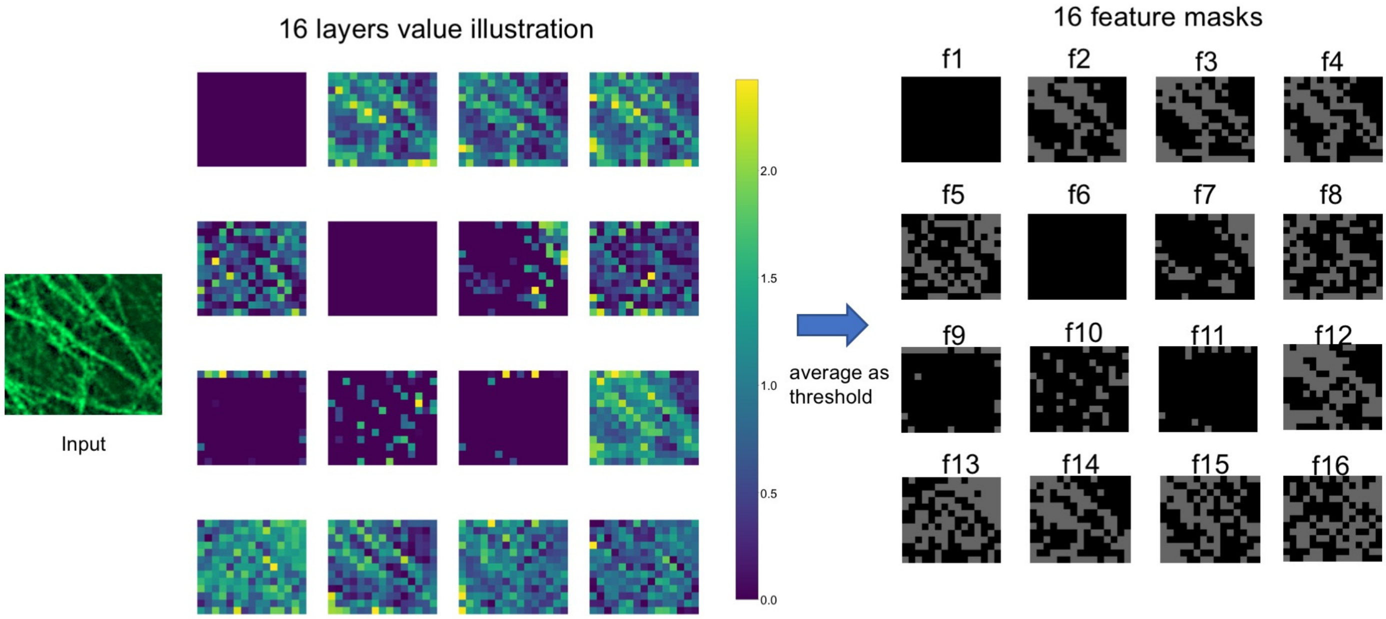

Figure S4: Generation of feature masks for representation of learned microstructural features of the hydrogels. The Keract[29] Python package was applied to compile outputs of the fifth layer (13×15×16) of the trained Autoencoder. Using the mean value of the 13×15 values at each feature extractor, we generated the corresponding 16 feature masks. The heatmap represents pixels of the input image that are extracted by the 16 feature extractors. The lighter areas represent the highlighted feature areas.
